# Economic impact and policy implications from urban shared transportation: The case of Pittsburgh’s shared bike system

**DOI:** 10.1371/journal.pone.0184092

**Published:** 2017-08-31

**Authors:** Konstantinos Pelechrinis, Christos Zacharias, Marios Kokkodis, Theodoros Lappas

**Affiliations:** 1 School of Computing and Information, University of Pittsburgh, Pittsburgh, PA, United States of America; 2 School of Business Administration, University of Miami, Miami FL, United States of America; 3 Carroll School of Management, Boston College, Boston MA, United States of America; 4 School of Business, Stevens Institute of Technology, Hoboken NJ, United States of America; Universitat Jaume I, SPAIN

## Abstract

During the last years the number of cities that have installed and started operating shared bike systems has significantly increased. These systems provide an alternative and sustainable mean of transportation to the city dwellers. Apart from the energy sustainability benefits, shared bike systems can have a positive effect on residents’ health, air quality and the overall condition of the currently crumbling road network infrastructure. Anecdotal stories and survey studies have also identified that bike lanes have a positive impact on local businesses. In this study, driven by the rapid adoption of shared bike systems by city governments and their potential positive effects on a number of urban life facets we opt to study and quantify the value of these systems. We focus on a specific aspect of this value and use evidence from the real estate market in the city of Pittsburgh to analyze the effect on dwellers’ properties of the shared bike system installed in the city in June 2015. We use quasi-experimental techniques and find that the shared bike system led to an increase in the housing prices (both sales and rental prices) in the zip codes where shared bike stations were installed. We further bring into the light potential negative consequences of this impact (i.e., gentrification) and discuss/propose two public policies that can exploit the impact of the system for the benefit of both the local government as well as the city dwellers.

## 1 Introduction

The age-old concept of cities as the paramount instrument of innovation and wealth creation has led to the unprecedented levels of urbanization that we have witnessed during the last decade. However, this comes with a price; cities have become the main source of crime, diseases and pollution, significantly deteriorating the quality of life of their inhabitants. One of the main factors contributing to air-pollution is the deep-rooted preference of city dwellers for private automobile mobility. Part of this preference is a result of the way planners designed cities in the mid 20th century, having in mind models such as the Radiant City of Le Corbusier. This resulted in inefficient or even complete lack of multimodal transportation systems.

Apart from the associated pollution, the increase in the number of automobiles in the streets leads to increased congestion and commute times which reduces the overall emotional health of dwellers. During the last years, urban planners have started re-thinking the mobility modes in a city and have come to realize that if we are to have a resilient, livable and sustainable urban environment we need to increase the diversity of urban mobility modes. This realization, in conjunction with the rapid advancements in mobile and ubiquitous computing that facilitates deployment and usage, has resulted in a number of cities worldwide replicating the successful experiment that started mainly in French cities in 2006, by adopting shared bike systems. These systems provide features of on-demand transportation similar to automobiles (in contrast to traditional public transit modes such as buses and the subway) while being at the same time a more energy efficient and healthy option. Of course, the practical range of this transportation mode is much more limited as compared to automobiles. Users can pick up and drop off the system’s bicycles from fixed docks around a city that they can identify through the web or a mobile application.

Despite the anecdotal claims for the potential environmental, economic and health benefits of shared bike systems (and alternative transportation modes in general) there is very little scientific evidence and studies to date to support them. For example, a recent study in Washington, DC [1], based on surveys with business owners (e.g., retail shops, restaurants etc.), reported that approximately 20% of them *perceived* a positive effect from the city’s shared bike system. Furthermore, a survey study from Oregon Transportation Research and Education Consortium [2] found that bike rider customers can spend more in the long run as compared to car drivers customers. While they spend less per trip, they also visit the establishments more often. In our work we aim at complementing similar studies by quantifying the *“value”* added from the shared bike systems on a specific aspect of urban life, namely, housing. We will not rely on the subjective perception and retrospective view of the involved parties, but rather we will utilize appropriate, objective, datasets. In particular, we examine the effect of a shared bike system on the real estate market by collecting the relevant data from the city of Pittsburgh. Home prices capture not only the value of the actual building but also the land value of the surrounding environment. Hence, they can provide a collective and objective representation of the importance of the shared bike systems for the city dwellers.

Having an objective valuation for the shared bike systems in various aspects of urban life is of great importance, since it can lead to educated policy making. For example, the existence of a direct impact on the real estate prices can be proven a burden to city dwellers in the long run due to the increased property taxes. This can lead, if local governments are not careful, to a new form of gentrification. Lower income families will be displaced from these areas and will have no access to the shared bike system. Note here also that, these are the families that most probably cannot afford the ownership of an automobile and hence, would benefit the most from affordable shared bike systems. Hence, policies need to be in place to avoid similar pitfalls. We elaborate on potential policies in Section 4.

The main contributions/findings of our work can be summarized in the following:

- We identify a clear positive effect of shared bike systems on the real estate market (in the city of Pittsburgh)
- We examine both the microscopic (within a city) as well as the macroscopic (between cities) effects on the real estate market
- We examine the impact of the shared bike system on the housing rental market in the city of Pittsburgh
- We propose policies that can exploit this positive impact for the benefit of both the local governments as well as the general population.

We would like to emphasize here that our conclusions are based on the analysis from the real estate market in the city of Pittsburgh. These conclusions are not necessarily transferable to other metropolitan areas. For instance, while our conclusions might hold for cities with the same population, demographics and geography, the installation of a similar system in a mega-city—e.g., NYC, Los Angeles etc.—might have much less impact on the real estate value, if at all. However, we expect our study to stimulate further and deeper examination of similar interventions.

### 1.1 Related studies

#### 1.1.1 Shared bike and bike lanes infrastructure

With the rapid deployment of shared bike systems around the globe a number of studies that examine the structure, operations and efficiency of these systems have appeared. Faghih-Imani *et al.* [3] study the re-balancing problem of the available bikes at the stations and provide a model that is able to minimize the re-balancing costs. Other studies utilize time-series analysis and models to estimate the state of the system and the “pulse” of the city [4–6]. The temporal properties of the shared bike systems [7, 8] further support the hypothesis that they are mainly used for commuting purposes.

Bike lanes (independent of the presence of a shared bike system) have also been the focal point in a number of studies. There are many anecdotal stories for the positive impact of bike lanes on local economy. For instance, the addition of bike lanes on Magnolia street at Fort Worth has been reported to have increased restaurant revenues along the street by 179% [9]. Similar anecdotal stories have been reported for other cities as well (e.g., Denver [10]), while a nice review of the economic benefits of bicycling is provided by Krizec [11].

Our work is complementary to the existing literature on bike systems and infrastructure and aims at identifying the impact of a shared bike system on the city dwellers’ properties in the city of Pittsburgh. A significant volume of literature that explores the connection between traditional modes of transportation and housing prices that we briefly review in what follows.

#### 1.1.2 Transportation and property values

The impact of transportation infrastructure on the housing market has been an important topic in the transportation literature. However, the vast majority of the existing research focuses on the traditional modes of transportation, such as subway, buses and light rail. For example, a number of studies have evaluated the impact of railway stations on property values. Dewees [12] found that the presence of a subway station increases rent prices within a radius of 1/3 mile. Similar findings have been reported in follow up studies (e.g., [13–15]), while other studies have reported insignificant or even negative impact on the neighboring real estate (e.g., [16]). This variability in the results can be attributed to a variety of reasons [17]. For example, the different studies focus on different areas, with different characteristics and different types of railway. In particular, the meta-analysis by Debrezion *et al.* [18] shows that the impact of commuter rail is much higher as compared to that of light and heavy rail. In another meta-analysis Ryan [19] shows that the results vary in a predictable manner based on the way that the accessibility to transport stations is measured (i.e., whether the physical distance or the travel time is used). While these studies examine the impact of the existing infrastructure, more recently, Efthymiou and Antoniou [20] using data from Thesaloniki in Greece, evaluate the impact of a planned, i.e., not yet functional, subway line on the sales and rent prices of houses. Even though rent prices are not significantly affected the sales prices are negatively impacted, potentially due to the negative externalities associated with the construction phase that is projected to last for 5 years. In another direction, Diaz and McLean [21] identify an additional way that property values near light railway systems can benefit in the long term. In particular, the rail accessibility will make the developement in the area attractive, which will in turns increase the land and property value.

Complementary to the existing studies, we focus on an alternative and sustainable mode of transportation, namely biking. Similar types of transportation are central to the new urbanism movement that has gained momentum the last few years and hence, it is crucial to evaluate their effects.

## 2 Materials and methods

In this section we will first describe the types of datasets that we collected for our study as well as, the difference-in-differences method upon which we will build our analysis.

### 2.1 Pittsburgh bike share system

**Fig 1:**
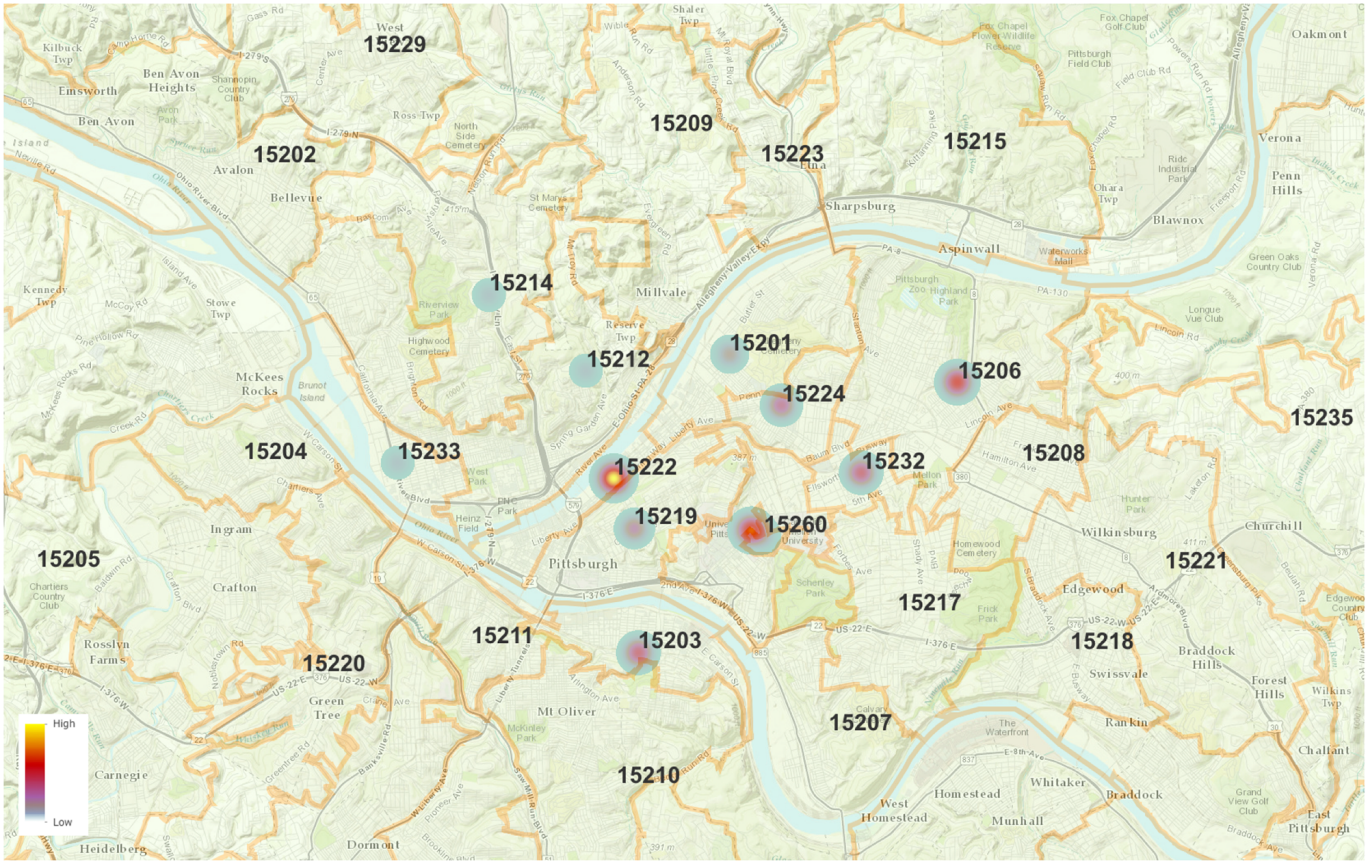
The non-uniform spatial distribution of the stations provides a setting that will allow us to examine the microscopic effects on real estate at the zip code level.

The city of Pittsburgh has installed 50 shared bike stations, with an average capacity of 15 bikes per station and a total of 500 bikes that started operating in June 2015. These stations are installed in 12 different zip codes and started operating during the same time. Within the first year of operation (but outside of our analysis period), 3 of the original stations have been re-located from their original position. The distribution of the stations is not uniform, with some zip codes having more infrastructure than others, with a median of 3 stations per zip code. Fig 1 depicts a heatmap with the spatial distribution of the shared bike stations at the zip code level from part of the Pittsburgh metro area (zip code borders are marked with orange lines). For example, zip code 15222 has the largest number of stations, 12 in total, and corresponds to the downtown Pittsburgh area. Users can pick up a bike from any station and drop it off to any other station. Users are charged either on a per ride-basis or through a subscription model that provides unlimited rides of 30 or 60 minutes duration.

**Fig 2:**
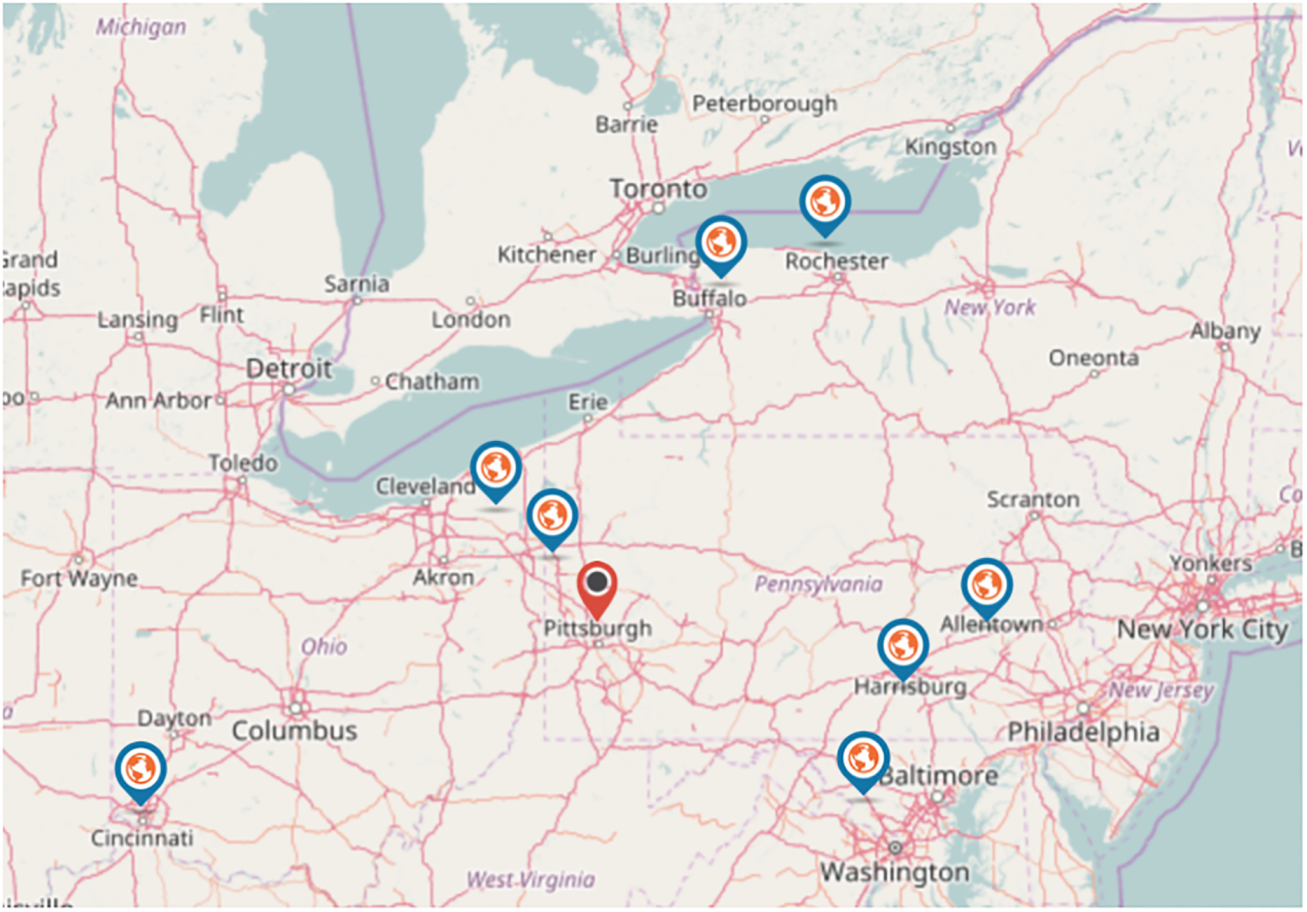
The cities we used for comparison are within driving distance to the city of Pittsburgh in order to ensure exposure to similar geographical dynamics to the extend possible.

Even though we have observational data, the installation process of the system in Pittsburgh allows us to evaluate its effect in the city’s real estate in a manner that resembles an experimental study. In particular, the market in the city of Pittsburgh has been exposed to the “treatment” of bike share system, while at the same time other similar metropolitan areas have not, thus serving as the control for our study (while we elaborate more in the following section Fig 2 presents the cities we used for comparison. Furthermore, supplementary material S1 Text presents relevant demographic information). Despite the fact that we did not randomly assign the treatment (i.e., decide on which city to install the system), the assignment process itself that governs the exposures arguably resembles a random assignment, since each one of these metropolitan areas could have installed a system as well. This is exactly the setting of a natural experiment that can pave the way beyond simple correlation analysis. Furthermore, the fact that bike stations have not been installed in all zip codes in Pittsburgh metro area is crucial for our study. Another important point is that our discussions with the share bike system operator revealed that there was not any particular planning that went in the initial deployment phase with regards to the locations that the stations were installed (other than making sure the network is *connected*). While we do not expect that this process is an exact replication of a randomized experiment—since certainly there are cognitive biases that lead humans make choices by subconsciously considering specific parameters—this process, in conjunction with some of our experimental design choices discussed later, should minimize any correlations between our dependent variable and other omitted variables from our model. This experimental setting allows us to compare the real estate valuation between areas in the Pittsburgh region where the transportation system has been affected by the shared bike system and areas where it has not. Simply put we will be able to examine the microscopic effects (i.e., at the zip code level) of the newly installed bike system on the real estate value.

### 2.2 Real estate Zillow data

In order to evaluate the effect of the shared bike system on the real estate market we collected data from Zillow, an online real-estate database that provides information about real estate sales and estimates of homes in the United States. Zillow also provides historic information dating back to 1996 on a monthly basis. Zillow provides the Zillow Home Value Index (ZHVI) calculated using both public and user-submitted data through a proprietary formula. Similarly, Zillow provides information about the rental properties, through the Zillow Rent Index (ZRI).

For our study we collect the following information:

- Time-series data for the ZHVI for all homes in 10 selected metro areas (including Pittsburgh) the choice of which is described in Section 3.
- Time-series data for the ZHVI and ZRI in the zip codes within Pittsburgh metro. We collect different time-series for all homes as well as, 1, 2, 3, 4 and more than 5 bedroom dwelling units separately.

At the zip code level, there is some missing information from the Zillow dataset. In particular, the time-series for 3 out of the 12 zip codes that include shared bike docks, are missing from the Zillow dataset. This might be due to various reasons, such as lack of information for past transactions in the area etc.

### 2.3 Difference in differences

The difference in differences (DD) [22] is a quasi-experimental technique that aims in identifying the effect of an intervention using observational data. The difference-in-differences method has been extensively used in the (applied) econometrics literature for making causal inferences from observational data and quantifying the impact of various public policies and interventions. For example, Greenwood and Wattal [23] used DD to quantify the impact of Uber operations on the drunk driving related homicides in two cities in California, while Chan and Ghose [24] studied the impact of the introduction of classified ads on the transmission of HIV. Using the same methodology, Chevalier and Mayzlin [25] studied the impact of book reviews on sales. In this work, we are using the difference-in-differences method to estimate the impact of the installation of shared bike system on the real-estate market, examining two specific hypotheses that we will develop in the following section.

DD requires observations obtained in different points in time, e.g., *t*_1_ and *t*_2_ (*t*_1_ < *t*_2_), for both the control (e.g., *o*_*c*,1_ and *o*_*c*,2_) and the treatment (e.g., *o*_*τ*,1_ and *o*_*τ*,2_) groups. The treatment group is exposed to the intervention only during *t*_2_. The difference between *o*_*τ*,2_ and *o*_*c*,2_ does not only include the effect of the intervention but it also includes other “intrinsic” differences between the two groups. The latter can be captured by their difference during time *t*_1_, i.e., *o*_*τ*,1_ − *o*_*c*,1_, where the treatment group has not been exposed to the intervention. The DD estimate is then:

$$\begin{array}{c} \delta_{\tau ,c}=\left(o_{\tau ,2}-o_{c,2}\right)-\left(o_{\tau ,1}-o_{c,1}\right) \end{array}$$ (1)

This removes any biases in the comparison during *t*_2_ between the treatment and the control group that could be the result from (i) permanent differences between those groups, as well as (ii) biases from comparisons over time in the treatment group that could be the result of trends.

The exactly same estimate for the DD can be formally derived through a linear regression that models the dependent variable *o*. In particular, we have the following model:

$$\begin{array}{c} o_{ilt}=\alpha_{l}+\beta_{t}+\delta \cdot D_{lt}+\epsilon_{ilt} \end{array}$$ (2)

where *o*_*ilt*_ is the dependent variable for instance *i* (at time *t* and location *l*), *α*_*l*_ and *β*_*t*_ are binary variables that capture the fixed effects of location and time respectively, *D*_*lt*_ is a dummy variable that represents the treatment status (i.e., *D*_*lt*_ = *α*_*l*_ ⋅ *β*_*t*_) and *ϵ*_*ilt*_ is the associated error term. The coefficient *δ* captures the effect of the intervention on the dependent variable *o*. It is then straightforward to show that the DD estimate $\hat{\delta }$ is exactly Eq (1). In particular, if ${\bar{o}}_{lt}$ is the sample mean of *o*_*ilt*_ and ${\bar{\epsilon }}_{lt}$ is the sample mean of *ϵ*_*ilt*_, using Eq (2) we obtain:

$$\begin{array}{cc} \left({\bar{o}}_{00}-{\bar{o}}_{01}\right)-\left({\bar{o}}_{10}-{\bar{o}}_{11}\right)= & \delta \left(D_{00}-D_{01}\right)-\delta \left(D_{11}-D_{10}\right)\\ & +{\bar{\epsilon }}_{00}-{\bar{\epsilon }}_{01}+{\bar{\epsilon }}_{10}-{\bar{\epsilon }}_{11} \end{array}$$ (3)

Taking expectations and considering the i.i.d. assumptions for the errors for the ordinary least squares we further get:

$$\begin{array}{c} E\left[\left({\bar{o}}_{00}-{\bar{o}}_{01}\right)-\left({\bar{o}}_{10}-{\bar{o}}_{11}\right)\right]=\delta \left(D_{00}-D_{01}\right)-\delta \left(D_{10}-D_{11}\right) \end{array}$$ (4)

**Fig 3:**
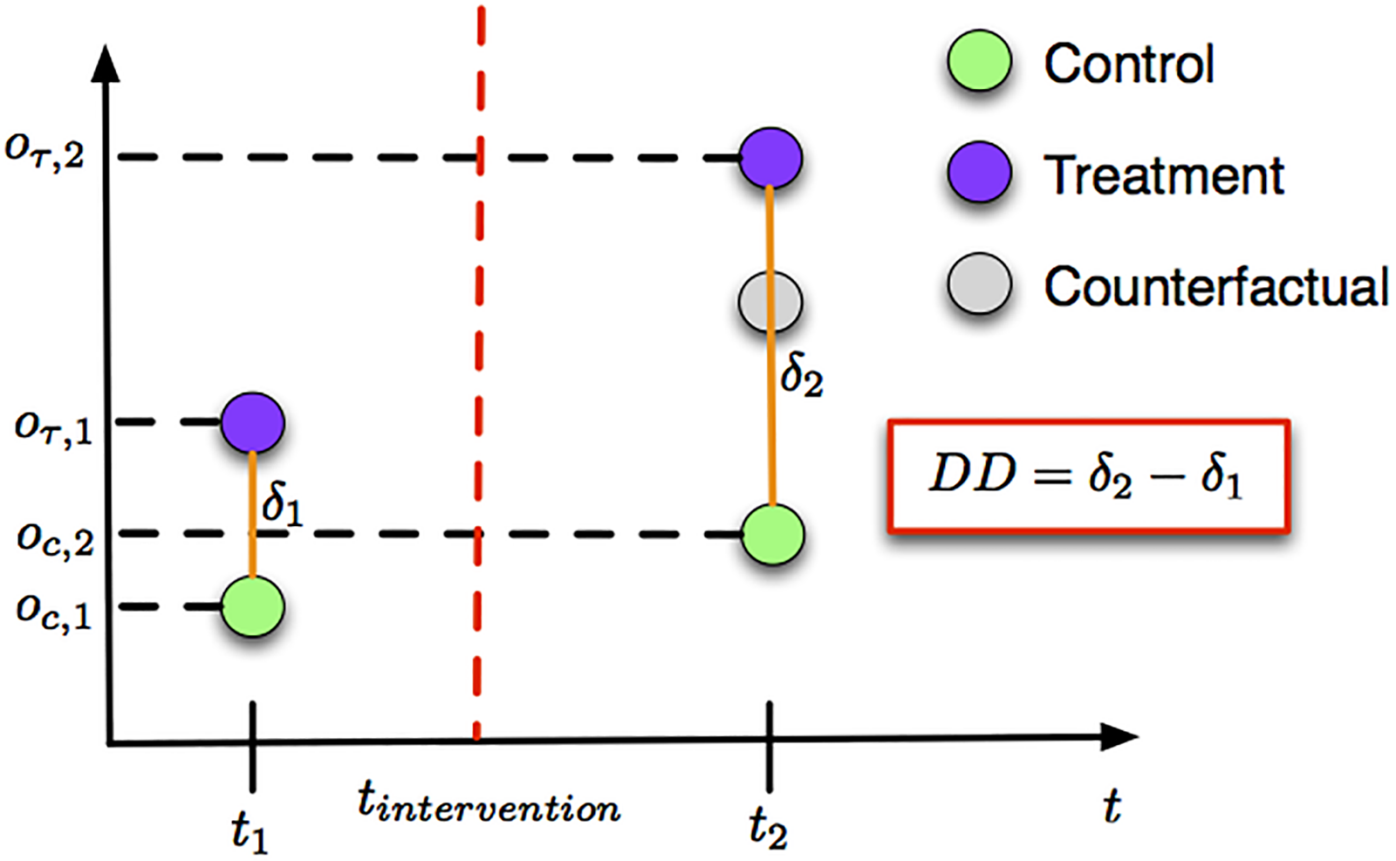
The difference-in-differences method.

Given that the dummy variable *D* is equal to 1 only when *l* = 1 and *t* = 1 (i.e., for the treatment group after the intervention), we finally get for the DD estimator:

$$\begin{array}{c} \hat{\delta }=\left({\bar{o}}_{00}-{\bar{o}}_{01}\right)-\left({\bar{o}}_{10}-{\bar{o}}_{11}\right) \end{array}$$ (5)

which is essentially the same as Eq (1). Therefore, one can estimate the DD using either of the Eqs (1) or (2). Fig 3 further depicts the estimation process. In order for the conclusions of a difference-in-differences analysis to be robust, the **parallel trend assumption** needs to hold. This assumption essentially states that the average change in the control group represents the counterfactual change expected in the treatment group if there was no treatment. Simply put, if there was not any treatment applied, we would have: (*o*_*τ*,2_ − *o*_*c*,2_) = (*o*_*τ*,1_ − *o*_*c*,1_), that is, the two groups would have a stable difference. This assumption is crucial for the conclusions to hold and many times overlooked when the method is applied. However, we formally show in Section 3 that the parallel trend assumption holds in our dataset.

The control and treatment groups/subjects are defined based on the spatial granularity of our analysis. In our study we are interested in both the macroscopic as well as the microscopic effects of the shared bike systems. Hence, in the first case, our treatment group is the whole city of Pittsburgh and the observation is the median housing price in the city, while the control group involves neighboring cities—with similar population and certainly below 1 million dwellers—that have not installed shared bike systems during the period covered by our study. We are focusing on neighboring cities (of similar population) in order to assure, to the extent possible, that the cities compared are subject to similar externalities related with the geography. In the case of microscopic effects, the subjects are defined as urban areas at the zip-code level, i.e., every zip-code in the Pittsburgh metropolitan area is one subject. Furthermore, the treatment group is defined as the set of subjects, i.e., zip codes, that include at least one shared bike station within their borders.

One of the experimental setup decisions that we have to make is what exactly are the two time-points that we will examine. Given that the system was installed and started operating on June 2015, we consider as the “intervention” time observation to be July 2015, i.e., we observe the median real estate price during July 2015. While this choice might appear to be too soon, it reduces the probability that the property values capture the impact of other external events as well. Simply put, we expect this experimental choice to minimize the impact of any omitted variable. Furthermore, the details of the system were already known a few months prior to the official inauguration of the system and hence, the prices can actually reflect this knowledge and expectation as it progressed through this timeline [20]. Our pre-intervention time could be May 2015, i.e., one month before the installation of the system. Nevertheless, for the same reason, the May prices might reflect expectations from the bike share system. Therefore, we choose as our pre-intervention time period to be January 2015, a time where the details for the upcoming bike sharing system were vague and hence, could not have been reflected in the real estate valuations.

### 2.4 Research hypotheses

As mentioned earlier there have been several studies that have revealed the positive link between (commuter’s) public transportation accessibility and housing property value [12–15, 18, 21]. At the same time there is various anecdotal evidence that point to a positive impact of biking infrastructure (e.g., bike lanes) on local businesses and economy. Hence, in this work we combine these findings in the literature to develop a research hypothesis that aims into examining the impact of shared bike systems on city dwellers’ property values. Furthermore, the sales and rental markets exhibit different dynamics and are targeting different population demographics. Current literature also has differentiated between the two markets when examining the link between transportation and real estate. For example, Landis *et al.* [26] explored the impact of BART on housing sales and rentals separately, while Efthymiou and Antoniou [20] showed that the impact of upcoming transportation upgrades is different on sales and rental values. Hence, we decide to examine the respective impacts separately. In particular, we will examine the following two hypotheses.

**Hypothesis 1** [Impact on Dwelling Units Values]: *The presence of shared bike transportation infrastructure impacts positively the housing values of nearby properties*.
**Hypothesis 2** [Impact on Rental Price]: *The presence of shared bike transportation infrastructure leads to an increase in the prices of nearby rental properties*.

In order to support or reject Hypotheses 1 and 2 we will rely on the ZHVI and ZRI data from Zillow respectively, utilizing the difference-in-differences method described earlier. We will further examine the presence of an impact on different spatial scales, that is, microscopically (i.e., intra-city) and macroscopically (i.e., inter-city).

## 3 Results

In this section we present our results and analyze the impact of the shared bike system at the city of Pittsburgh both from a macroscopic as well as a microscopic point of view using the difference-in-differences method detailed in the previous section.

### 3.1 Hypothesis 1

We start by comparing the housing values in the city of Pittsburgh with those in other similar, nearby cities. We then focus within the city of Pittsburgh by comparing the property values within different zip codes based on the presence of shared bike stations.

#### 3.1.1 Macroscopic effects

At a macroscopic level we are interested in examining what happened in the real estate prices in the city of Pittsburgh, prior and after the installation of the system, as compared to the case of other *similar* cities that did not have a bike share system installed during this period. In our comparison we are focusing on cities that are within 4 to 5 hours of drive from the city of Pittsburgh and that have similar characteristics. As we can see in supplementary material S1 Text, the cities picked have similar median age (standard deviation 1.9 years) and income (standard deviation 6K), while they are all medium to moderately small sized in terms of their population. Their geographic proximity and their demographic similarities increases our confidence that these cities are potentially affected by the same environmental and local economy externalities. To reiterate it is important that the cities we compare with did not receive a similar treatment during the period of the study (they could have installed earlier, but not during the time span we analyze).

Table 1 depicts the difference-in-differences estimate (Eq (1)) for every city. As we can see the average difference-in-differences between the city of Pittsburgh and the cities in the control group is *δ*_*Pittsburgh*,*c*_ = $850 (*p*-value <0.05). Simply put, the operation of shared bike system in the city of Pittsburgh, increased the median real estate price by an extra $850 on average as compared to other nearby, similar cities, which did not get a similar treatment. These provides initial evidence for the positive impact of the shared bike system on the housing value.

**Table 1:** The difference-in-differences between Pittsburgh and nearby similar cities implies a positive effect from the shared bike system.

City	Difference-in-differences
Allentown, PA	2,300
Baltimore, MD	700
Buffalo, NY	1,900
Cincinnati, OH	-100
Cleveland, OH	-100
Harrisburg, PA	1,100
Rochester, NY	200
Youngstown, OH	1,200
**p-value**	**0.03**

#### 3.1.2 Microscopic effects

In this part of our analysis we are interested in examining the effects of shared bike systems within the city of Pittsburgh with zip code being our spatial unit for analysis. Let us denote the median housing value within the *i*^*th*^ treated zip code, i.e., the *i*^*th*^ zip code that got shared bike stations, with *z*_*τ*,*i*_. We further denote with *z*_*c*,*j*_ the median housing value of the *j*^*th*^ control subject, i.e., the median housing value of the *j*^*th*^ Pittsburgh metro zip code that did not get any shared bike station installed. Finally, the set of all treated zip codes is denoted with T, while the one including all control zip codes is denoted with C.

We begin by examining the overall effect by performing the regression analysis of Eq (2). To reiterate, coefficient *δ* captures the average effect of the bike shared system on the dependent variable, i.e., the real estate value. Our regression analysis results in *δ* = 2985 (*p*-value <0.1), i.e., on average the presence of shared bike stations adds approximately an extra $3,000 value on average as compared to the areas without the docks, or approximately a 2.5% increase. For further validation of this effect we perform the following experiment.

Having established the presence of an effect at an aggregate level, we delve into the details for every zip code. In particular, for every treated zip code i∈T we calculate the average difference-in-differences with the control zip codes:

$$\begin{array}{c} {\bar{\delta }}_{i}=\frac{\sum_{j\in C}\delta_{i,j}}{\left|C\right|} \end{array}$$ (6)

With Δ_*i*_ being the dataset obtained from the difference-in-differences between treated zip code *i* and the control zip codes, i.e., $\Delta_{i}=\left\{\delta_{ij},\;\forall j\in C\right\}$, we can further perform the following test:

$$\begin{array}{cc} H_{0}: & {\bar{\delta }}_{i}=0 \end{array}$$ (7)

$$\begin{array}{cc} H_{1}: & {\bar{\delta }}_{i}>0 \end{array}$$ (8)

Note here that we perform the one-sided test since this is the direction of interest for our hypotheses. Table 2 presents the results for each of the treated zip-codes. As we can observe, the (average) difference-in-differences is positive for all the zip-codes that were subjected to the intervention. Furthermore, this average is statistically significant, at (least at) the 1% significance level, in *ϕ*_*τ*_ = 88% of the treated subjects. Only exception is the zipcode 15212, where while ${\bar{\delta }}_{15212}>0$, the corresponding p-value is 0.16. However, note here that, 15212 received a small treatment *level* with only one shared bike station in 15212. This single shared bike station might not be enough to generate a significant positive impact on the housing properties within 15212. In the supplementary material (S2 Text) we further examine the significance of the value of *ϕ*_*τ*_ through a null experiment.

**Table 2:** The average DD for all the treated subjects is positive (and statistically significant) leading to the conclusion that the shared bike system has a positive impact on the real estate valuation. Significance codes: 0 ‘***’ 0.01 ‘**’ 0.05 ‘*’ 0.1 ‘.’ 1 ‘’.

Zip Code	${\bar{\delta }}_{i}$ ($)
15206	7,374***
15203	874**
15214	2,474***
15212	374
15213	4,474***
15219	2,274***
15222	1,074***
15232	5,674***
15233	2,274***

**Fig 4:**
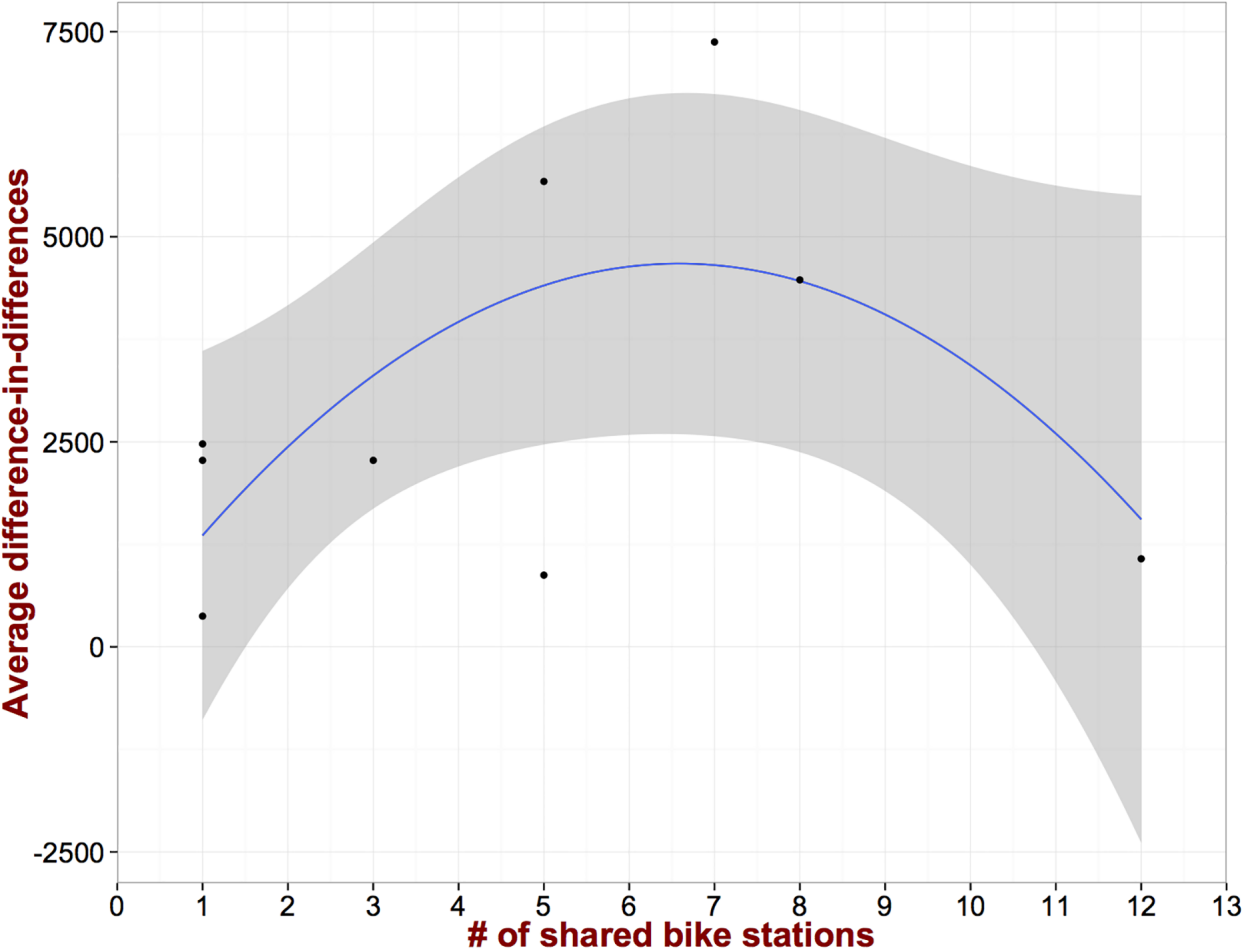
Increasing the number of shared bike stations within a zip code beyond a threshold, might have counter-intuitive results.

An interesting aspect of our dataset is the fact that we can define different levels of intervention *λ*_*i*_ for every treated zip code i∈T based on the number of bike stations that were installed. As aforementioned there are zip codes that have just one station, while there are other zip codes that have as many as 12. Fig 4 depicts ${\bar{\delta }}_{i}$ as a function of the intervention level *λ*_*i*_. As we can see for small *λ*_*i*_, increasing the treatment leads to an increase on the effect. However, beyond a specific level (in our dataset this corresponds to *λ* = 7), the effect reduces, even though it remains positive. One of the potential reasons for this behavior can be the ***perception*** of city-dwellers and home owners/buyers that additional bike stations remove *valuable* curb parking spots from the already limited supply. Given the high value of a parking spot [27], it is possible that reducing their number will affect negatively the perception of the real estate value of the area. Unfortunately, regardless of how plausible this explanation appears, our data cannot further support or reject this hypothesis and it is only a speculation at this point.

Finally, we break down the impact of shared bike system on different types of housing. Table 3 presents the average difference-in-difference for every zip code and housing type. For some specific combinations of zip codes and housing type Zillow does not provide any information, potentially due to the small number of this dwelling type in the specific zip code. Given that it is also possible that individual zip codes include a small sample size of each dwelling unit and hence, the corresponding coefficient might be biased even if it is statistically significant (e.g., think of a zip code with only five houses of a specific dwelling type unit for calculating the median housing value). Therefore, in order to obtain more robust and meaningful results, we focus on the last row of the table, where we present the average difference-in-differences coefficient for each type of dwelling unit over all the zip codes As we can see the positive impact is clear on almost all types of dwellings. However, there is one case where the average difference-in-differences is negative (at the significance level of *α* = 0.1). This corresponds to dwelling units with at least 5 bedrooms. In hindsight, one might have expected this, since the owners of such big dwellings are typically people that rely on auto-mobiles for their commute needs. Hence, they do not see any value on a shared bike system. In fact, Young and Jones [28], analyzing census data reveal that car ownership is significantly increased for dwelling units with 5 or more bedrooms. Furthermore, as alluded to above, these dwellers might actually perceive the presence of the stations as negative in the sense of reduced parking supply. However, overall, the positive impact of the system on Pittsburgh’s real estate market is indubitable from our analysis in the section.

**Table 3:** Owners of large houses (5 and more bedrooms) appear to not value the presence of a shared-bike system since the average difference-in-differences for these type of houses is negative. Owners of such types of dwellings rely on auto-commute and hence, they might view bike stations as harmful (e.g., reducing parking availability). The significance codes correspond to the hypothesis test described by Eqs (7) and (8): 0 ‘***’ 0.01 ‘**’ 0.05 ‘*’ 0.1 ‘.’ 1 ‘’.

Zip Code / House Type	1 Bed	2 Beds	3 Beds	4 Beds	5+ Beds	Condos	Single Family
15206	8,175***	2,838***	5,216***	8,659***	-7,654***	8,060***	6,509***
15214	N/A	2,638***	1,216***	-1,340**	-2,454	4,060***	2,209***
15212	-525	2,138***	316	-1,740***	-3,454*	2,860***	-90
15213	3,175***	1,238***	6,616***	3,759***	-13,954***	2,960***	5,309***
15219	11,875***	2,738***	-983***	2,359***	-3,954*	3,260***	3,009***
15222	-625	-9,661***	N/A	N/A	N/A	360	N/A
15232	1,575***	5,538***	4,716***	20,259***	4,645**	-339	21,509***
15233	N/A	N/A	916***	5,059***	N/A	2,160***	309
**All (average)**	3,941*	**1,066**	2,573*	5,287*	**-4,469**.	2,922**	5,537*

#### 3.1.3 Parallel trend assumption

As mentioned in Section 3 in order for the difference-in-differences method to be able to provide reliable results and conclusions, the parallel trend assumption needs to be satisfied. In order to test whether this assumption is satisfied in our dataset, we can compute the difference-in-differences between the treated and the control groups for earlier time periods that do not include the installation of the bike system (i.e., the treatment). If the computed difference-in-differences is insignificant, i.e., *δ* = 0 for all statistical purposes, then the parallel trend assumption holds [29].

In what follows we examine the parallel trend assumption for both the microscopic and macroscopic analyses. In particular, we will examine the difference-in-differences coefficient at the zip code level as well as at the city level. For applying DD, we choose exactly the same period but one year back (i.e., 01/2014-07/2014) as well as the second half of 2014 (i.e., 07/2014-12/2014). Tables 4 and 5 presents the average difference-in-differences for the two time periods for both the zip code and city level.

**Table 4:** The average difference-in-differences for the treated zip codes for two selected periods without the shared bike system intervention. The overall DD coefficient is not significant for either of the time periods examined. Significance codes: 0 ‘***’ 0.01 ‘**’ 0.05 ‘*’ 0.1 ‘.’ 1 ‘’.

Zip Code	01/2014-07/2014	07/2014-12/2014
15206	−5,297***	−2,263***
15203	11,102**	9,236***
15214	−6,897***	−1,863***
15212	−5,697***	−663**
15213	4,402***	2,836***
15219	−3,997***	−669**
15222	11,202***	−11,363***
15232	19,402***	13,436***
15233	3,802***	136
**p-value**	**0.3**	**0.7**

**Table 5:** The parallel trend assumption is satisfied at the macroscopic level as well. The inter-city difference-in-differences coefficient is insignificant for the periods preceding the treatment.

Zip Code	01/2014-07/2014	07/2014-12/2014
Allentown, PA	-3,900	-1,600
Baltimore, MD	-100	2,500
Buffalo, NY	100	600
Cincinnati, OH	-800	-900
Cleveland, OH	300	900
Harrisburg, PA	100	3,300
Rochester, NY	900	1,600
Youngstown, OH	1,500	2,000
**p-value**	**0.7**	**0.11**

As we can see from the results half of the treated zip codes exhibit positive difference-in-difference during these reference periods, while the rest exhibit negative. In other words, the fraction of zip codes exhibiting positive difference-in-difference is equal to the one that might have been expected just by chance. The corresponding *p*-values for testing whether the average coefficient *δ* is different than zero, conclude that *δ* is insignificant. The same is true for the macroscopic analysis, where as we can see from Table 5 the inter-city DD coefficient is insignificant. This result further strengthens our conclusions for a positive effect of the bike shared system on the real estate prices, since the crucial parallel trend assumption for the difference-in-differences methods holds in our setting.

Given that we have observational data, the above analysis is essentially rejecting the possibility of reverse causation. In particular, even though as alluded to above there was not any detailed planning that went into the initial deployment of the system, it could still be the case that the increasing trend in the real estate market within specific zip-codes (or cities) subconsciously led the operator to install shared bike system in specific locations. Therefore, the observed positive average difference-in-differences might have been related with some latent external factors that led to an increase in the housing prices in these areas, which then led the share bike system operator choose the shared bike station locations. However, the results from our analysis reject this possibility, since prior to the treatment the average difference-in-differences was 0 for all practical purposes. Hence, prior the installation of the system there was not an increasing differential trend in the specific zip codes that could have been a subconscious factor for the operator for installing the shared bike docks (since to reiterate the operator has verified to us that there was not a focused study for the locations of the docks).

We would like to emphasize here that while overall there is not any trend identified with regards to the difference-in-differences, individual zip codes (or cities) exhibit non-zero difference-in-differences. Of course, one should not expect to obtain a coefficient exactly equal to 0 in order to conclude that there is not any trend. The housing market has an inherent volatility that contributes towards this observations. Furthermore, these non-zero coefficients can potentially capture the impact of other externalities (irrelevant to the shared bike system) that were present during the examined period. However, despite these possibilities when considering an individual zip code (or city), overall there is not any trend, providing us with important evidence for the robustness of our conclusions.

#### 3.1.4 Diagnosis of standard errors

As discussed by Bertrand *et al.* [30] apart from heterogeneous pre-treatment trends, one of the most significant potential problems with the difference-in-differences method is that of correlations within the residuals. Prior work [23, 30, 31] has relied on random treatment models as a robustness check. This random implementation model determines the probability of the observed effect occurring purely by chance. This placebo test allows us to clearly identify (i) if correlation within spatial units (i.e., cities or zip codes) is unaccounted for, and (ii) the extent to which changes in the housing values may be occurring in untreated locations, or the effect of the bike treatment is driven by a single—or few—location, essentially providing a check agains outliers.

**Fig 5:**
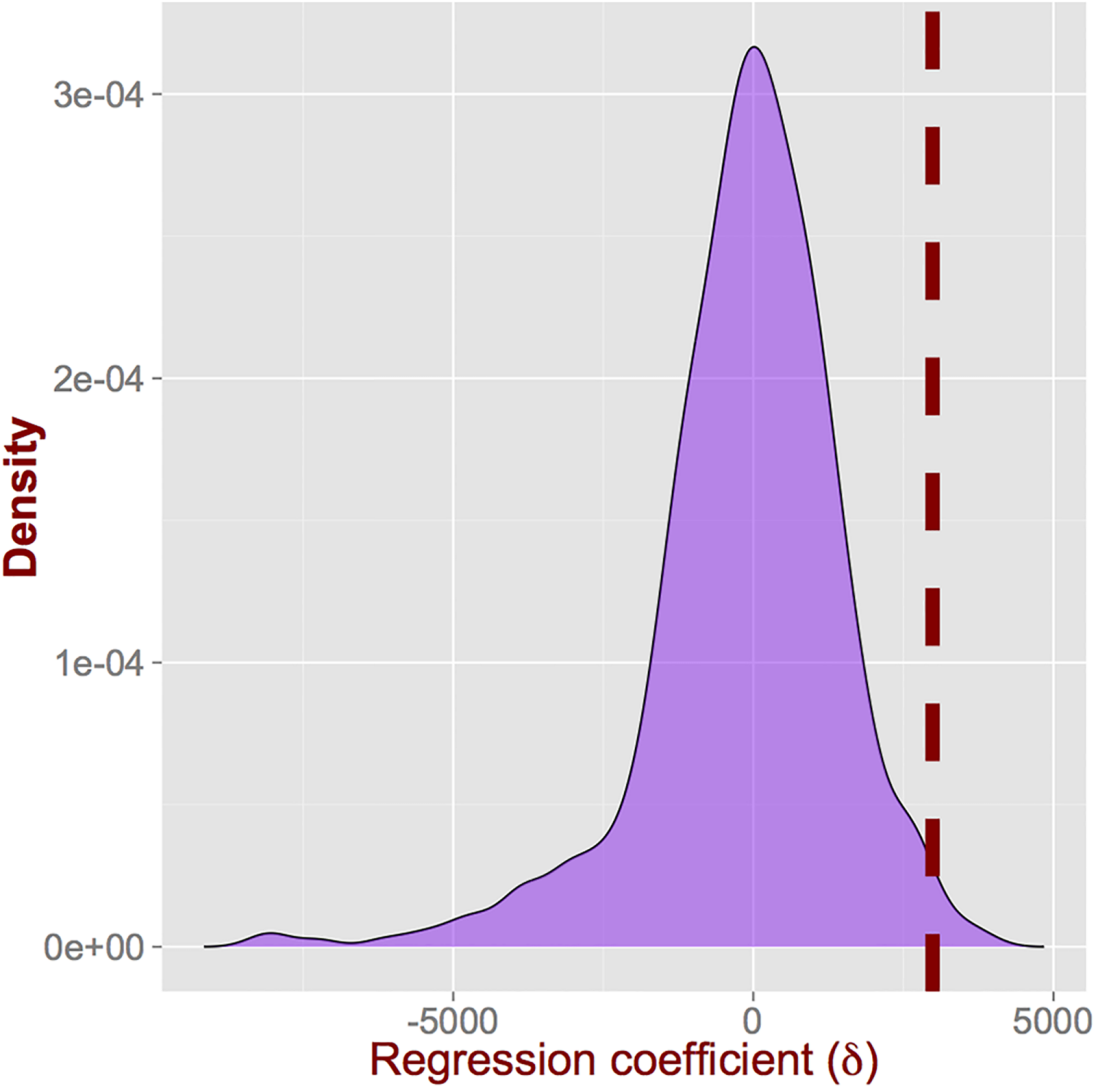
The difference-in-differences regression coefficient with random assignment of treatment provides an *effect* centered around 0 (and slightly skewed to the left), strengthening our conclusions for the impact of shared bike systems.

In our macroscopic experiment we assigned randomly the treatment to the different cities considered and calculated the placebo difference-in-differences coefficient. Our results indicate that we cannot reject the null hypothesis that the coefficient is not zero. For the microscopic experiment, we assign randomly the treated areas (equal to the number of treated areas in the real-world setting) and we keep the rest of the zip codes as our randomized controls. To reiterate, from this randomization we can obtain an estimate of the coefficient *δ* expected by *chance*. By repeating this process B times (in our case we set B=500) we can obtain an estimate of the probability of observing a value equal or greater than *δ* = 2985, i.e., the one from the real setting, by chance. Fig 5 depicts the distribution of *δ* obtained from our experiment. For comparison, we also mark the value of *δ* in the real setting with a vertical dashed line. The results for this experiment show that there is an exceptionally small probability (0.01) of the obtained coefficient to have been observed by luck, further strengthening the conclusion for the impact of the shared bike systems on the housing values. These results suggest that correlations within the city/zip codes have been accounted for.

### 3.2 Hypothesis 2

Until now we have focused on the actual value of the dwelling units. Nevertheless, a large part of the population is renting their housing and hence, it is important to identify any effect on rental properties as well. Following the same analysis as above we compute the average difference-in-differences for the rental properties in the treated zip codes. Table 6 depicts our results. As we can see the installation of the shared bike system leads to an increase in the rental prices as well, thus, capturing the value of the system for the house rental market. Even though not as strong as the effect on the actual property values, on average, i.e., over all the treated zip codes, the difference-in-differences is $26.5 (*p*-value < 0.05). This means that the additional increase in the rent price due to the presence of the shared bike system is $26.5. The same result was obtained through the linear regression model (2). Note here that the parallel trend assumption also holds for the rental prices (aggregate difference-in-differences coefficient for the pre-treatment periods examined were 0.11 and 0.13 respectively with p-value > 0.85). Furthermore, we performed the same placebo experiment as above, for the rental prices and we find that the value observed (i.e., $26.5) has a very small likelihood of having been observed by chance. In particular, the placebo difference-in-differences coefficient is insignificant ($12.5, p-value = 0.16).

**Table 6:** The operations of the shared bike system increases the rental values. Significance codes: 0 ‘***’ 0.01 ‘**’ 0.05 ‘*’ 0.1 ‘.’ 1 ‘’.

Zip Code	${\bar{\delta }}_{i}$ ($)
15206	24***
15201	25**
15214	3
15212	15**
15213	−8*
15219	−17**
15222	128***
15224	12**
15232	47***
15233	36***

**Fig 6:**
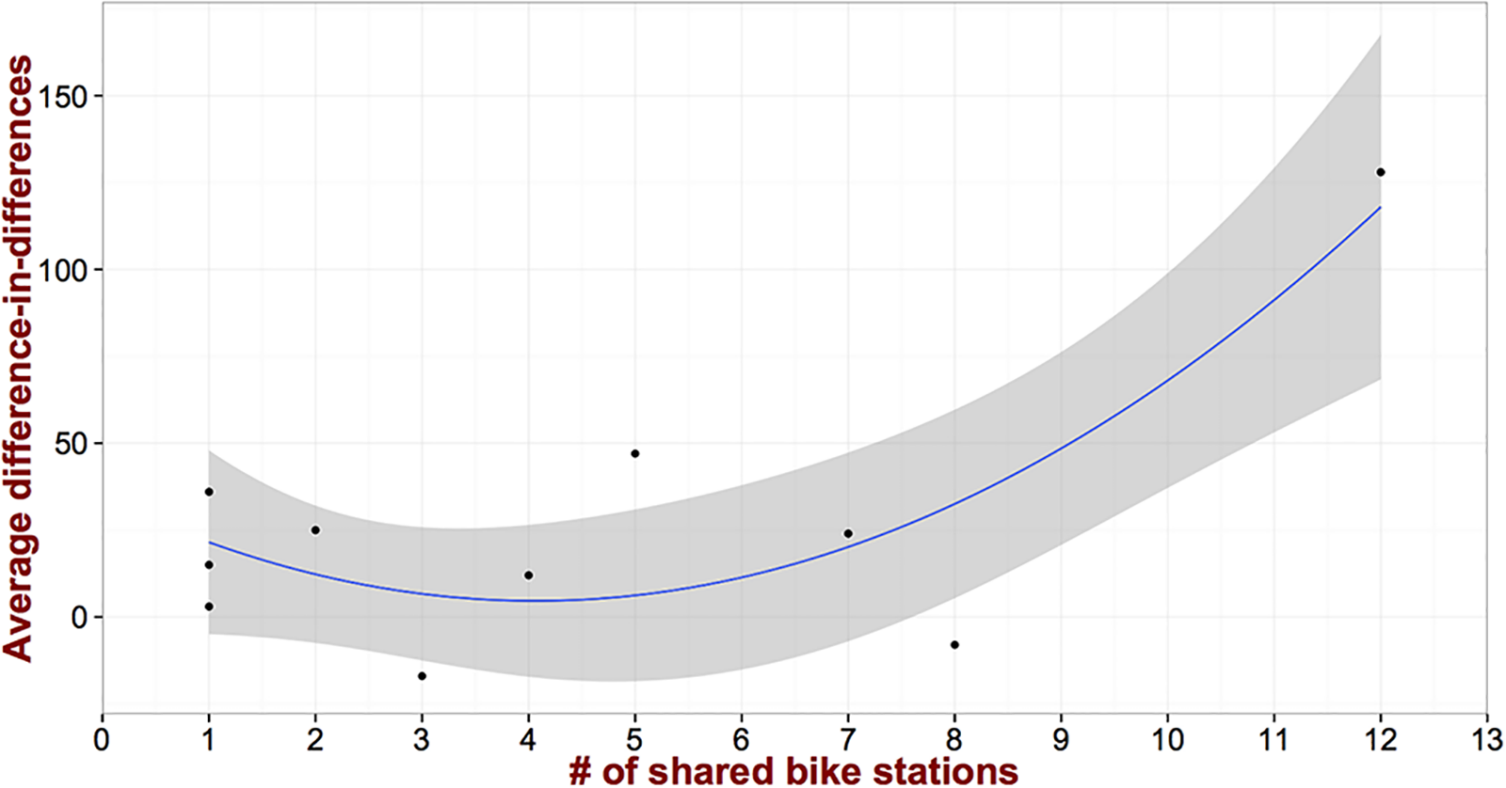
The average difference-in-differences for rental properties as a function of the number of shared bike stations exhibits different behavior as compared to dwelling sales price.

We further examine the relationship between the number of shared bike stations and *δ*. Fig 6 presents the results. As we can see the behavior is different compared to the dwelling sales price. More specifically, for small number of stations the effect is fairly constant. However, further increasing the capacity of the system leads to a large increase in the rental prices. This is in stark contrast with the pattern observed in Fig 4. This discrepancy can potentially be explained from the different demographics of people that rent and own their dwelling units respectively. In particular, renters tend to be college students and young professionals (two sizable demographics in the city of Pittsburgh) who are largely advocates of the new urbanism movement and value greatly alternative modes of transportation over cars [32]. On the contrary, and as alluded to above, home owners seem to perceive the presence of many bike stations less positive since they eliminate *valuable* curb parking spaces (detailed demographics are presented in supplementary material S3 Text). Of course, this does not mean that this increasing pattern will persist as we keep adding more bike stations. Furthermore, for each of the values of the independent variable of this relationship (i.e., the number of bike stations) we have very few observations (in most of the cases only one). This essentially means that the relationship identified might not be as robust as the aggregate difference-in-differences coefficient estimates. Note here that this also applied to the relationship presented in Fig 4.

We also performed a macroscopic analysis comparing the rent index of Pittsburgh with that of other similar cities (the same as in Table 1). The average difference-in-differences is $-16.5 with a p-value of 0.38, and hence, for all statistical purposes it cannot be deemed different than 0. Of course, given the fairly small sample size the statistical power of the test might not be enough to detect small, but significant, differences. Nevertheless, even though in a microscopic scale there appears to be an impact on rent values, our macroscopic results do not allow us to fully support Hypothesis 2.

One of the main reasons for the different effect on the property value and rent prices might be the different temporal dynamics of the two markets. In particular, rental prices do not alter as fast as property values can change. While individual houses are not sold as often, within a zipcod several properties are sold each month allowing for the market to react faster. On the contrary, typically rental prices have an annual-cycle and hence, re-adjustments of prices might be less immediate due to long-term leasing agreements that cannot be altered prior to their expiration. For example, the owner of a rental property that was occupied by a tenant during June 2015 with a lease valid through the end of 2015 could not adjusted adjusted the rent price earlier. On the contrary a home owner with her house on the market during June 2015, could potentially adjust the asking price if she wanted.

## 4 Discussion

The positive impact on the housing values is consequently translated to increased income for the local government due to increased property taxes. For example, the average property tax for the zip codes in Table 2 is approximately 3%. Hence, the local government of Pittsburgh will obtain on average an additional $2,985 ⋅ 0.03 ≈ $90 per dwelling unit within these zip codes. From this perspective, local governments can view similar shared transportation systems as investments that can be paid off fairly quickly through the increased property taxes.

Nevertheless, extreme care through appropriate policies needs to be taken from the local governments in order to avoid side effects that this new mode of transportation might cause. The areas with the shared bike stations—at least in Pittsburgh—are areas with medium to high income households on average. Hence, even a small increase in the housing prices and the corresponding property tax can have a significant effect in dislocating the parts of the population that belong to the lower layers of the income scale, or providing a *barrier-to-entry* for families that would like to relocate within the areas under consideration. Even when gentrification within a neighborhood does not happen (e.g., because the neighborhood itself is homogenous with respect to socio-economic factors), public/private investment in shared bike systems can potentially exacerbate the wealth gap between neighborhoods; the appreciation of the real estate in one area leads to more wealth to their owners and “rich-gets-richer” phenomena can appear. One could argue that higher property values increase the net worth of everyone including those in the lower layers of the income scale. While this is true this assets lack liquidity and hence, cannot immediately compensate for the increased cost of owning a house. The rising cost of housing is in general associated with lower consumer purchasing power, which further burdens the dwellers in the lower layers of the income scale.

Furthermore, the fact that the parallel trend assumption holds essentially means that the housing prices within the various zip codes in Pittsburgh exhibited homogeneous changes. Hence, even though the treatment and control zip codes can exhibit differences (one of which is the accumulated wealth), these differences did not reflect any difference in the changes of the real estate market in the corresponding neighborhoods. However, the installation of the shared bike system triggered this increase and it might have been a combination of the shared bike system and other differences between the neighborhood (which to reiterate had not lead to changes prior to the shared bikes) that is responsible for the increase in the real estate value. Simply put, the shared bike system might have just been the catalyst of the observed increase in the housing prices. In what follows we briefly provide the blueprint of two possible policies that can potentially avoid gentrification and further wealth gap increase. We also discuss possible technical difficulties and obstacles in their implementation.

### 4.1 Bike benefit districts

The additional income from the increased property taxes can be split into three funds, the *infrastructure*, the *development* and the *tax refund* funds. The infrastructure fund will contribute towards paying off the initial investment of the shared bike system, while the development fund will be returned back to the districts that generated this revenue. Of course, such a policy would require the main investment in the shared bike system to be public and not private. The distribution of the development fund can be either based on the number of shared bike stations, the population or even the number of shared bike system users per district. These proceeds will be further used for urban (re)development of these districts (e.g., new pavement, green spaces etc.). The tax refund fund will be used to subsidize the housing tax of lower income families within the district, essentially lowering the effective housing tax rate for these dwellers in an effort to avoid their displacement. Similar benefit districts (with the exception of the tax refund fund) have been created in a number of cities with respect to income generated from parking metered zones. The poster child for such parking benefit districts is Pasadena, CA, which managed to rejuvenate its downtown area solely through the revenue generated from parking meters [33]. However, contrary to parking benefit districts there are some important details that need to be considered in order for bike benefit districts to succeed. For instance, in the case of parking there is no ambiguity with regards to which districts is responsible for the parking revenue generated, that is, the district where the parking meter is located. However, in the case of a bike benefit district there are two stations involved; an origin one and a destination one, which can belong to different districts. Which district should get the benefit? Furthermore, while metered parking spaces are ubiquitous the same is not true for shared bike stations. Given this, dwellers of districts with no shared bike infrastructure can potentially feel disadvantaged, thus, creating unnecessary tensions. In general, while bike benefit districts is a simple idea that has a precedent at parking benefit districts, there are some technical details that need to be considered in order for a similar policy to work.

### 4.2 Gas emission urban trade market

One other way to potentially avoid the aforementioned gentrification phenomena, is through a *carefully* designed gas emission urban trade market. In particular, the local government will need to first identify the effect of the shared bike system on gas emissions. This can be done by using a methodology similar to the one used in our work and the appropriate dataset (e.g., particulate matter concentration). Consequently depending on the effect of each area in reducing the gas emissions, property tax allowances will be provided as an incentive for dwellers to adopt the use of the system. For the rental properties, subsidies can be provided for the dwellers that are user’s of the system as well. If carefully designed and operated this can have the potential to increase the revenue of the system, thus, (i) making it self-sustained, (ii) improving the quality of life in the city and (iii) avoiding gentrification effects. Nevertheless, a gas emission trade market in an urban scale does not come with out challenges. The most crucial one is how to accurately measure the gas emissions at such a small spatial scale. Furthermore, similar to the bike benefit districts, dwellers in areas without shared bike stations can be disadvantaged.

Overall, we believe that both suggested policies have the potential to (a) alleviate possible gentrification problems driven by the shared bike system, and/or (b) help with funds for financing public tasks in the corresponding areas. However, given that the focus of our work is not to analyze policy impacts or its feasibility, our presentation has been only at a high level. In order for the potentials of these (or other) policies to be realized a more detailed plan of implementation need to be laid out.

## 5 Conclusions

In this work we examine the effect of shared bike systems on the real estate market using data from the city of Pittsburgh. For our analysis we rely on the difference-in-differences method, a quasi-experimental technique. Our results indicate that there is a clear positive impact of this new mode of transportation on the housing prices. We further discuss the implications this can have both for the local governments (i.e., increased income through property taxes) as well as for the city dwellers (i.e., gentrification). We finally propose two specific policies that can exploit this effect for the common, social good of a city.

We would like to emphasize here that we only have observational data and hence, we are limited in what conclusions we can make. Furthermore, our experimental design allows us to make conclusions for the short term impact of the shared bike system in the city of Pittsburgh. In other words, we cannot claim that this benefits are sustainable in the long-term. Most importantly, given that our data are obtained from the city of Pittsburgh, more analysis is required in order to examine how generalizable and transferable our conclusions are. For instance, the positive impact on the real estate values might disappear in a large city such as NYC or a city that is not bike friendly (e.g., Los Angeles, Atlanta etc.). Hence, our study needs to be seen as a positive (case study) evidence for the value of shared bike systems. We hope that our work will stimulate further research on the economic benefits of sustainable and multimodal transportation modes.

As part of our future work, we opt to examine more cities and explore whether the results are generalizable and how potential differences on the observed effects can be explained. More importantly though, we plan to delve into the suggested policies in order to recommend solid solutions to avoid the possible negative externalities.

## Supporting information

S1 Text Demographic information. (PDF)

S2 Text Null experiment for *ϕ*_*τ*_. (PDF)

S3 Text Demographics of home owners and renters. (PDF)

## References

[1] Anderson R, Childs E, Doumi Y, Godard J, Chris Marshall AM, McConnell K, et al. Economic Impact and Operational Efficiency for Bikeshare Systems. Technical Report Virginia Tech University. 2014;.

[2] Clifton KJ, Muhs C, Morrissey S, Morrissey T, Currans K, Ritter C. Consuber Behavior and Travel Mode Choices. Technical Report OTREC. 2012;.

[3] Faghih-Imani A, Hampshire RC, Marla L, Eluru N. An Empirical Analysis of Bike Sharing Usage and Rebalancing: Evidence from Barcelona and Seville. Available at SSRN: http://ssrncom/abstract=2657197. 2015;.

[4] Kaltenbrunner A, Meza R, Grivolla J, Codina J, Banchs R. Urban Cycles and Mobility Patterns: Exploring and Predicting Trends in a Bicycle-based Public Transport System. Pervasive Mob Comput. 2010;6(4):455–466. 10.1016/j.pmcj.2010.07.002

[5] Jensen P, Rouquier JB, Ovtracht N, Robardet C. Characterizing the speed and paths of shared bicycle use in Lyon. Transportation Research Part D: Transport and Environment. 2010;15(8):522–524. 10.1016/j.trd.2010.07.002

[6] Froehlich J, Neumann J, Oliver N. Sensing and Predicting the Pulse of the City Through Shared Bicycling. In: Proceedings of the 21st International Jont Conference on Artifical Intelligence. IJCAI’09; 2009. p. 1420–1426.

[7] Murphy E, Usher J. The Role of Bicycle-sharing in the City: Analysis of the Irish Experience. International Journal of Sustainable Transportation. 2015;9(2):116–125. 10.1080/15568318.2012.748855

[8] O’Brien O, Cheshire J, Batty M. Mining bicycle sharing data for generating insights into sustainable transport systems. Journal of Transport Geography. 2014;34:262–273. 10.1016/j.jtrangeo.2013.06.007

[9] Blue E. Bikenomics: How Bicycling Can Save the Economy Bicycle Series. Microcosm Publishing; 2013.

[10] type;. Available from: https://nextcity.org/daily/entry/bike-lanes-help-salt-lake-city-economy.

[11] Krizec KJ. Estimating the economic benefits of bicycling and bicycle facilities: An interpretive review and proposed methods In: Essays on transport economics. Springer; 2007 p. 219–248.

[12] Dewees DN. The effect of a subway on residential property values in Toronto. Journal of Urban Economics. 1976;3(4):357–369. 10.1016/0094-1190(76)90035-8

[13] Damm D, Lerman SR, Lerner-Lam E, Young J. Response of Urban Real Estate Values in Anticipation of the Washington Metro. Journal of Transport Economics and Policy. 1980;14(3):pp. 315–336.

[14] Grass RG. The estimation of residential property values around transit station sites in Washington, D.C. Journal of Economics and Finance. 1992;16(2):139–146. 10.1007/BF02920114

[15] Voith R. Transportation, Sorting and House Values. Real Estate Economics. 1991;19(2):117–137. 10.1111/1540-6229.00545

[16] Gatzlaff DH, Smith MT. The Impact of the Miami Metrorail on the Value of Residences near Station Locations. Land Economics. 1993;69(1):pp. 54–66. 10.2307/3146278

[17] Debrezion G, Pels E, Rietveld P. The Impact of Rail Transport on Real Estate Prices: An Empirical Analysis of the Dutch Housing Market. Urban Studies. 2011;48(5):997–1015. 10.1177/0042098010371395

[18] Debrezion G, Pels E, Rietveld P. The Impact of Railway Stations on Residential and Commercial Property Value: A Meta-analysis. The Journal of Real Estate Finance and Economics. 2007;35(2):161–180. 10.1007/s11146-007-9032-z

[19] Ryan S. Property Values and Transportation Facilities: Finding the Transportation-Land Use Connection. Journal of Planning Literature. 1999;13(4):412–427. 10.1177/08854129922092487

[20] Efthymiou D, Antoniou C. Measuring the effects of transportation infrastructure location on real estate prices and rents: investigating the current impact of a planned metro line. EURO Journal on Transportation and Logistics. 2014;3(3-4):179–204. 10.1007/s13676-013-0030-4

[21] Diaz RB, Mclean VA. Impacts of rail transit on property values. In: American Public Transit Association Rapid Transit Conference Proceedings; 1999.

[22] Ashenfelter O, Card D. Using the Longitudinal Structure of Earnings to Estimate the Effect of Training Programs. The Review of Economics and Statistics. 1985;67(4):648–60. 10.2307/1924810

[23] Greenwood BN, Wattal S. Show me the way to go home: an empirical investigation of ride sharing and alcohol related motor vehicle homicide. The review of Economics and Statistics. 2015;.

[24] Chan J, Ghose A. Internet’s dirty secret: assessing the impact of online intermediaries on HIV transmission. Chan J, and Ghose A, “Internet’s Dirty Secret: Assessing the Impact of Online Intermediaries on HIV Transmission”, MIS Quarterly. 2013;38(4):955–976.

[25] Chevalier JA, Mayzlin D. The Effect of Word of Mouth on Sales: Online Book Reviews. Journal of Marketing Research. 2006;43(3):345–354. 10.1509/jmkr.43.3.345

[26] Landis J, Cervero R, Guhathukurta S, et al. BART at 20: property value and rent impacts. prepared for the 74th Annual Metting of the Transportation Research Board. 1995;.

[27] Shoup D. The High Cost of Free Parking. American Planning Association; 2011.

[28] Young A, Jones P. Residential Parking—Quantity and Quality. In: Transport and Development Planning; 2005.

[29] Autor DH. Outsourcing at will: The contribution of unjust dismissal doctrine to the growth of employment outsourcing. Journal of labor economics. 2003;21(1):1–42. 10.1086/344122

[30] Bertrand M, Duflo E, Mullainathan S. How much should we trust differences-in-differences estimates? The Quarterly journal of economics. 2004;119(1):249–275. 10.1162/003355304772839588

[31] Donald SG, Lang K. Inference with difference-in-differences and other panel data. The review of Economics and Statistics. 2007;89(2):221–233. 10.1162/rest.89.2.221

[32] Gallini J. Demographics and Their Relationship to the Characteristics of New Urbanism: A Preliminary Study. Texas State University-San Marcos; 2010.

[33] Kolozsvari D, Shoup D. Turning small change into big changes. Access Magazine. 2003;1(23).

