# Supplementary material for: Economic impact and policy implications from urban shared transportation: The case of Pittsburgh’s shared bike system
Source: PLoS One. 2017 Aug 31;12(8):e0184092. doi: 10.1371/journal.pone.0184092 (PMC5578620; doi:10.1371/journal.pone.0184092)
Supplement: S1 Text — (PDF) [file pone.0184092.s001.pdf]

S1 Text. Demographic Information. In the following tables we present the median age and income for each of the treated zip codes in the city of Pittsburgh. We also provide their classification based on ESRI's tapestry system (`http://www.esri.com/landing-pages/tapestry`). Tapestry classifies US zip codes into 67 distinct segments based on demographics and socioeconomic characteristics. This tapestry label fuses several information beyond just age and income. While some zip codes might be assigned several (weighted) labels, we report the top one for each zipcode. More detailed information can be obtained at: `http://www.esri.com/data/esri_data/ziptapestry`, where the interested reader can see an interactive map.

**Zip Code**	Median Age	Median Income ($)	ESRI Tapestry
15206	37.9	35K	Modest Income Homes
15203	30.4	38K	College Towns
15214	39.2	38K	Rustbelt Traditions
15212	40.3	35K	Set to Impress
15213	23.5	20K	College Towns
15219	25.9	18K	Social Security Set
15222	32.3	45K	Metro Renters
15232	29.2	47K	Metro Renters
15233	37.9	30K	Old & Newcomers
15224	35.2	29K	Set to Impress
15201	39.2	35K	Set to Impress
15260	19	33K	Retirement Community & Dorms to Diplomas

Table 1: Demographics for the treated zip codes in the city of Pittsburgh. Rows in blue font are those for which we do not have Zillow data for.

We further present in Table 2 the demographic information for the different metro areas used in our analysis. These data were obtained from `www.city-data.com`, which compiles data from several government resources.

**City**	Median Age	Median Income ($)	Population
Allentown, PA	32.7	37K	119,104
Baltimore, MD	34.7	44K	622,793
Buffalo, NY	33.1	33K	258,703
Cincinnati, OH	32.7	35K	298,165
Cleveland, OH	36	29K	389,521
Harrisburg, PA	32.8	34K	49,082
Rochester, NY	31.4	32K	209,983
Youngstown, OH	37.5	24K	65,062
Pittsburgh, PA	32.9	41K	305,412

Table 2: Demographics for the cities we used for comparison.
