# Supplementary material for: Economic impact and policy implications from urban shared transportation: The case of Pittsburgh’s shared bike system
Source: PLoS One. 2017 Aug 31;12(8):e0184092. doi: 10.1371/journal.pone.0184092 (PMC5578620; doi:10.1371/journal.pone.0184092)
Supplement: S2 Text — (PDF) [file pone.0184092.s002.pdf]

S2 Text. Null Experiment for $\phi_{\mathcal{T}}$.

To further delve into the above results we perform the following null experiment. In particular, we randomly assign "pseudo" treatment to the actual control zip codes $j \in \mathcal{C}$. Then for these pseudo treatment groups we will perform the same analysis as above and examine what fraction ϕ exhibits a positive average difference-in-differences (at the significance level $\alpha = 0.05$). We repeat the above process $\mathcal{B} = 500$ times. The reason for this null experiment is to establish a baseline for the expected ratio ϕ of positive average difference-in-differences for randomly selected zip codes. Figure 1 presents the boxplot of the obtained fraction ϕ for our null experiment along with an horizontal (blue) dashed line that represents $\phi_{\mathcal{T}}$. As it becomes evident the baseline obtained from the null experiment for ϕ is significantly lower (p-value ≈ 0) and in fact close to the "random mark" of 0.5, further strengthening our finding for the positive link between shared bike system and housing prices.

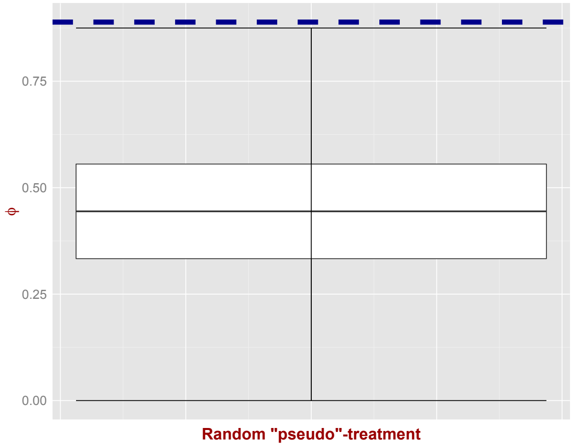

Figure 1: Randomly assigned "pseudo" treatments indicate that the baseline value for ϕ is significantly lower than the one observed in the actual treatment subjects (dashed blue line).
