# Supplementary material for: Economic impact and policy implications from urban shared transportation: The case of Pittsburgh’s shared bike system
Source: PLoS One. 2017 Aug 31;12(8):e0184092. doi: 10.1371/journal.pone.0184092 (PMC5578620; doi:10.1371/journal.pone.0184092)
Supplement: S3 Text — (PDF) [file pone.0184092.s003.pdf]

S3 Text. Demographics of Home Owners and Renters.

According to US Census American Community Survey the demographic characteristics of home owners and renters are very different (`http://eyeonhousing.org/2014/04/characteristics-of-owners-and-renters/`). In particular, with regards to the **age** of tenants, the majority of home owners fall between the ages of 45 to 54, while for the renters the group that includes the majority of renters is 25 to 34 years of age. Furthermore, there is a significant difference with regards to the **median annual income** of home owners and renters. In particular, the median income for rent-occupied households was $31,888, while that for owner-occupied households was $65,514. Moreover, 26.1% of the renter-occupied dwelling units are single-income households, while only 13.3% of owner-occupied units are single-income households. Finally, the largest share of owner-occupied units is married households at 60.1%, while for renter-occupied units the majority of the households are non-family (47.1%).
